# Assessing the Impact of a Health Education Intervention for Post-secondary International Students in Canada

**DOI:** 10.7759/cureus.42243

**Published:** 2023-07-21

**Authors:** Yasir H Khan, Shivajan Sivapalan, Sijia Wang

**Affiliations:** 1 Family Medicine, University of Toronto, Toronto, CAN; 2 Campus Health Centre, Ontario Tech University, Oshawa, CAN

**Keywords:** stress, covid-19, university health, college health, mental health, sexual health, international student, international student health, student health, health education

## Abstract

Objective

The objective is to determine the impact of a health education intervention on self-rated health knowledge, levels of stress and anxiety, and ability to find and access school resources for international students studying at a Canadian University and College.

Participants and setting

This is a pre- and post-intervention survey. Undergraduate and graduate international students on the shared campus of Durham College and Ontario Tech University in Oshawa, Ontario were included.

Interventions

International students participating in this study received two structured visits, scheduled two weeks apart. At the start of the first visit, students completed a baseline survey which included questions on self-rated health knowledge, stress levels, and ability to access school resources. At this visit, students received 30 minutes of structured health education from a registered nurse on the topics of sexual health and adult immunizations.

At the second visit, students received 30 minutes of structured teaching from the registered nurse on the topics of mental health, COVID-19, and campus resources. Upon the completion of this teaching, students completed a post-intervention survey with the same questions as the pre-survey, to gauge for changes related to the intervention.

Results

T-values were calculated for each survey item from the pre and post-survey. These t-values were used as the outcome measure to determine changes in health knowledge, stress levels, and ability to access resources following the intervention. In total, there were 202 participants. Statistical analysis showed significant t-values for all survey items in the pre- and post-analysis. Following the education intervention, the highest t-values were noted in self-rated sexual health knowledge (t-value 16.80, p < 0.001), ability to find and access school resources (t-value 16.14, p < 0.001), and current level of stress/anxiety in regard to being in a new country (t-value 14.04, p < 0.001).

Conclusion

Following a structured health education intervention, international students reported significant increases in self-rated health knowledge for specific topics, ability to find and access school resources, ability to get help for a mental health issue, and significant decreases in self-rated stress/anxiety. These results can support further exploration of health education in international student populations to ensure these students are adequately informed and supported when arriving in a new country.

## Introduction

The international student population has experienced tremendous growth in Canada over the past 15 years. In 2008, international students represented 6.4% of the post-secondary student population and consisted of just over 100,000 students [1]. By 2019, the population of international students had tripled to 318,000 which represented 16.2% of all post-secondary students and accounted for a 57.2% growth in post-secondary program enrollments [1]. In Canada, international students predominantly originate from India and China; in 2019 students from India accounted for 30% of all issued foreign study permits in Canada, while students from China accounted for 24% of study permits [2]. More recently, students from India accounted for 41% of all foreign study permits issued in 2022, while students from China accounted for 9% [2].

While dealing with the rigors and pressures of achieving academic success, international students simultaneously face the added psychological and physical stress of adapting to a new country with differing cultural and social norms [3-5]. Having left their original social support networks in their home country, it can be quite difficult for students to establish relationships and support systems in a new country [4]. The inability to adapt to acculturative stress during this transition has been shown to be detrimental to the international student’s physical and mental health, leading to challenges with anxiety, confusion, depression, homesickness, loneliness, stress, sleeping difficulties and more [3,4,6]. This is further exacerbated by the experience of racial microaggressions; south and east Asian international students in Canada have experienced social exclusion, derision for their accent and language proficiency, negative and inaccurate stereotypes, and insensitivity to their cultural perspectives and needs [6]. Research has also found that higher levels of unmet interpersonal needs, specifically feelings of belongingness or burdensomeness, were associated with increased suicidal ideation amongst international students [7]. Ethnic discrimination, feelings of entrapment, and the inability to escape unbearable situations all further elevated international students’ sense of emotional distress [7].

A study led by the World Health Organization (WHO) noted that the vast majority of post-secondary students with clinically significant mental health disorders remain untreated or inadequately treated despite the availability of a variety of efficacious treatment options [8,9]. A follow-up study by WHO identified that post-secondary students hesitate to seek help as they prefer to handle their issues alone or with the support of friends and relatives, highlighting that attitudinal barriers may be more influential than structural barriers [10]. International students were found to be even less likely than domestic peers to seek help due to cultural factors, negative stigma, linguistic barriers, and limited awareness of existing mental health services [4,11,12]. Those that do seek help are more likely to seek it from university counseling services and religious leaders than from general practitioners, psychologists, or community mental health services compared to domestic peers [4]. These studies highlight the overall need for culturally sensitive mental health education and services tailored to the unique needs of international students.

Additionally, international students would likely benefit from structured education around sexual health. Prior studies suggest that Asian international students generally had lower levels of sexual health literacy compared to domestic peers [13,14]. While studies note that international students are less likely to engage in high risk sexual activity, sexually transmitted infection (STI) testing rates are also much lower [13,15]. A 2007 subnational youth survey in India found that only 49% had positive knowledge of nonterminal contraception methods, and only 28% of young women had any comprehensive awareness of HIV [16]. In addition, the overall acceptance rate for pap smear screening in India remains low at only 8.3% [17]. In China, only 25.7% of Chinese women ages 20-64 years old had a pap test in 2015 [18]. These figures contrast with the 74% of women in Canada between the ages of 25-69 years old who had up-to-date pap tests in 2017 [19]. According to WHO data, the age-standardized rate of incidence of cervical cancer is approximately 5.5 cases per 100,000 women in Canada, compared to 18.0 per 100,000 women in India, and 10.7 per 100,000 women in China [20].

Mental health and sex education can vary significantly in different countries. It is crucial to approach education around these topics in a culturally sensitive manner by providing appropriate literature, as well as opportunities for students to ask questions or raise concerns in a non-judgmental, welcoming, and inclusive environment.

The objective of this research study was to assess the impact of an educational intervention for international students on their self-rated ability to access resources, their health-related stress, as well as their health knowledge. Limitations in health knowledge in the international student population can negatively affect their adjustment to a new country through increased stress and decreased use of available resources; the goal of this study is to show that these issues can be positively impacted through structured health education. There is currently limited literature examining the impact of health education in addressing health-related knowledge gaps in this population. These gaps must be addressed to empower new international students in their education journey and to ensure they are aware of healthcare resources available to them in helping to adjust and thrive under stressful circumstances in a new country.

Of note, this was part of a larger overall research study looking at multiple areas of international student health, including gaps in immunization, cervical cancer screening, and the incidences of specific medical conditions including thyroid disorders, iron deficiency, Vitamin B12 deficiency, and anemia. The findings of these other aspects of the research study will be discussed in future publications.

## Materials and methods

Procedure

Eligible international students from Ontario Tech. University and Durham College (OTU/DC) were registered in this study between May 27, 2020 and May 27, 2022. Recruitment consisted of a presentation about the research study at orientation events for new international students held in September and December 2020 and 2021. In addition, flyers were posted in the Campus Health Centre, and handouts regarding the study were provided to international students attending the Campus Health Centre for other services. Interested participants were provided contact information for the clinic to book the first of two appointments at the OTU/DC Campus Health Centre. 

At the initial visit participants completed a questionnaire that collected basic demographic data including gender, age, and country of origin. Further data collected on this questionnaire included past medical history, allergies, medications, alcohol/tobacco/recreational drug use, diet and exercise patterns, and sexual health screening. The participants were also required to complete a pre-intervention survey that consisted of self-evaluation questions to assess the participants’ knowledge regarding mental health, sexual health, and COVID-19, as well as current stress levels related to their health, being in a new country, and to COVID-19, and finally, their ability to access school resources and whether they would know what to do if they needed to seek help for a health issue. After completion of the pre-survey, participants met with a doctor to review their health concerns and for a focused physical examination. Once this was completed, students were offered a routine panel of bloodwork which screened for iron deficiency, thyroid disorders, anemia, and Vitamin B12 deficiency, as well as serology to check for immune status to vaccine-preventable diseases (hepatitis A/B, measles, mumps, rubella, and varicella). This bloodwork was part of the larger research protocol, the results of which will be discussed in future publications. Depending on the history provided or specific concerns of the student, other tests were included if clinically relevant (i.e., STI screening).

The participant then had a one-on-one structured education session with a female registered nurse. At this first visit, the discussion and teaching centered around a scripted list of topics including sexual health and adult immunizations. Sexual health topics included safe sex practices, the importance of consent, the use of contraception methods, the signs and management of STIs, and the benefits of pap smears for appropriate patients. Immunization education centered around reviewing the risks and benefits of vaccination against vaccine-preventable diseases, as well as the risks and complications of those diseases, including hepatitis, measles, mumps, rubella, chicken pox and more. Students were also provided teaching during this component on the human papillomavirus vaccine, including benefits for males and females for receiving this vaccine series. This initial session lasted for 30 minutes and included opportunities for participants to ask questions, or to request further detail on topics that were more individually relevant. At the end of the first session, the participant was provided a package with various educational resources that they could review during the two-week interval between visits. These resources provided further information on mental health, nutrition, hygiene, sexual health, and various resources available on campus.

Following the initial visit, students were booked for a follow-up appointment at a two-week interval. At this visit, participants had an opportunity to discuss any further concerns from their initial visit or any question they may have had after reviewing the resource package. At this time, the physician would also review any relevant test results with the participant. Results were entered into an electronic medical records-based data tool to track relevant blood work. Any medical interventions which were initiated based on the results were also tracked in this tool (e.g., initiation of thyroid replacement therapy, iron supplements). The participants then had a second 30-minute visit with a registered nurse on the same day to receive medical education on mental health, COVID-19, campus resources as well as general information about their insurance coverage. Mental health topics included on-campus mental health supports such as medical services, mental health nursing, coaching, psychiatry, and crisis services, as well as online support programs, and community crisis and addictions programs. Teachings around COVID-19 included signs and symptoms to look for, how to protect oneself from contracting it, how it is transmitted, and when to seek COVID-19 testing or further medical attention. Campus resource and insurance education focused on providing students with information on what was covered by their insurance plans including dental, vision, physiotherapy, massage, and more. The importance of good dental care was also reviewed during this visit.

After the conclusion of the second educational session, the participants completed a post-intervention survey. This contained the same self-evaluation questions as the pre-survey to assess any changes in their health knowledge, stress level, and ability to access resources following the intervention.

Statistical analysis

For statistical analysis, paired t-tests were conducted using IBM SPSS Statistics software (Statistical Package for the Social Sciences Version 29.0.1.0 (IBM Corp., Armonk, NY)) to determine if there was a significant difference in students' survey responses before and after the educational intervention. This statistical testing was chosen due to the pre- and post-nature of our intervention, which all 202 participants in the study completed. A p-value of <0.05 indicates statistical significance. Responses from the survey were converted to a 5-point Likert scale, with the exception of one question which assessed whether students would know what to do if they needed help for a mental health issue; for these questions, the pre-and post-response options were “yes,” “no,” and “maybe,” so the responses to this question were analyzed as a 3-point Likert scale.

## Results

In total, there were 202 participants in this research study, 109 female (54%) and 93 Male (46%). One hundred fifty-nine students were registered students with Durham College (79%) and 43 from Ontario Tech University (21%). Participants had an average age of 25 years with an age range of 17-50 (Table 1).

**Table 1:** Mean age of study participants

n	Minimum	Maximum	Mean	Std. Deviation
202	17	50	25.0297	5.13006

The vast majority of international students enrolled in the study were from India (n=129, 63.9%). Other common countries of origin included the Philippines (n=11, 5.44%), Nigeria (n=9, 4.46%), Iran (n=9, 4.46%), and Sri Lanka (n=7, 3.47%) (see Table 2 for further information on countries of origin of study participants).

**Table 2:** Country of origin of study participants

Country of Origin	Number of Participants	Percent of Total Participants
India	129	63.90
Philippines	11	5.44
Iran	9	4.46
Nigeria	9	4.46
Sri Lanka	7	3.47
Jamaica	6	2.97
Pakistan	4	1.98
Colombia	4	1.98
China	3	1.49
Bangladesh	3	1.49
Honduras	2	0.99
El Salvador	2	0.99
Trinidad and Tobago	2	0.99
Vietnam	2	0.99
Indonesia	1	0.50
Ecuador	1	0.50
Panama	1	0.50
Nepal	1	0.50
Ghana	1	0.50
Barbados	1	0.50
Uganda	1	0.50
Brazil	1	0.50
Zimbabwe	1	0.50
Total	202	

Statistical analysis with paired t-test indicates a significant difference in all of the items measured on the pre and post intervention survey. These results are summarized in Table 1. The most significant difference in post-intervention responses were seen in students self-rating of sexual health knowledge (pre-intervention survey mean Likert score = 3.48, post-intervention survey mean Likert score = 4.06, t-value=16.80, p < 0.001), and their self-rating on ability to find and access school resources (pre-intervention survey mean Likert score = 3.53, post-intervention survey mean Likert score = 4.09, t-value=16.14, p < 0.001). Table 3 summarizes the pre- and post-intervention self-rating scores by participants for each survey item. Table 4 summarizes the paired t-test statistical analysis results.

**Table 3:** Mean self-rating scores by participants for pre- and post-intervention survey questions *Due to the response options this item was scored as a 3-point Likert scale

Survey Items	Pre / Post	Mean	n	Std. Deviation	Std. Error Mean
How would you rate your knowledge about mental health ? (Very Poor = 1, Very Good =5)	Post	3.9851	202	0.8194	0.05765
	Pre	3.505	202	0.90465	0.06365
How would you rate your knowledge about sexual health ? (Very Poor = 1, Very Good =5)	Post	4.0644	202	0.79226	0.05574
	Pre	3.4802	202	0.84176	0.05923
How would you rate your knowledge about the COVID-19 Pandemic ? (Very Poor = 1, Very Good =5)	Post	4.3564	202	0.6704	0.04717
	Pre	3.9752	202	0.70843	0.04984
How would you rate your current level of stress/anxiety when thinking about being in a new country ? (Very stressed =1, Not stressed =5)	Post	3.6337	202	1.08562	0.07638
	Pre	3.1386	202	1.18052	0.08306
How would you rate your current level of stress/anxiety when dealing with your own health ? (Very stressed =1, Not stressed =5)	Post	3.5248	202	1.07522	0.07565
	Pre	3.2376	202	1.04761	0.07371
How would you rate your current level of stress/anxiety regarding the COVID-19 Pandemic? (Very stressed =1, Not stressed =5)	Post	3.7673	202	1.12428	0.0791
	Pre	3.5446	202	1.10201	0.07754
If you had to get help for a health or mental health issue at Durham College or Ontario Tech. University, would you know what to do? (No = 1, Maybe = 2, Yes = 3)*	Post	2.505	202	0.83008	0.0584
	Pre	2.0743	202	0.90297	0.06353
How would you rate your ability to find and access school resources for yourself ? (Very Poor = 1, Very Good =5)	Post	4.0941	202	0.80178	0.05641
	Pre	3.5297	202	0.99332	0.06989

**Table 4:** Statistical analysis from paired t-tests of pre- and post-intervention survey responses

Survey Items	Paired Differences					t-value	Degrees of Freedom	Significance	
	Mean (Post - Pre)	Std. Deviation	Std. Error Mean	95% Confidence Interval of the Difference				One-Sided p	Two-Sided p
				Lower	Upper				
How would you rate your knowledge about mental health ?	0.4802	0.50085	0.03524	0.41071	0.54968	13.627	201	< .001	< .001
How would you rate your knowledge about sexual health ?	0.58416	0.49409	0.03476	0.51561	0.65271	16.803	201	< .001	< .001
How would you rate your knowledge about the COVID-19 Pandemic ?	0.38119	0.48689	0.03426	0.31364	0.44874	11.127	201	< .001	< .001
How would you rate your current level of stress/anxiety when thinking about being in a new country ?	0.49505	0.50122	0.03527	0.42551	0.56459	14.038	201	< .001	< .001
How would you rate your current level of stress/anxiety when dealing with your own health ?	0.28713	0.46439	0.03267	0.2227	0.35156	8.788	201	< .001	< .001
How would you rate your current level of stress/anxiety regarding the COVID-19 Pandemic?	0.22277	0.41714	0.02935	0.1649	0.28065	7.59	201	< .001	< .001
If you had to get help for a health or mental health issue at Durham College or Ontario Tech. University, would you know what to do?	0.43069	0.6599	0.04643	0.33914	0.52225	9.276	201	< .001	< .001
How would you rate your ability to find and access school resources for yourself ?	0.56436	0.49707	0.03497	0.49539	0.63332	16.136	201	< .001	< .001

## Discussion

Key findings of our study included significant positive differences in self-ratings by international students following educational intervention for all measured areas of health knowledge, stress, and ability to navigate resources. The largest differences in pre and post-intervention responses were found in students’ self-ratings of their ability to find and access school resources, their sexual health knowledge, their current level of stress about being in a new country, and their mental health knowledge. Overall, these findings support the utilization of a health-education program with international students in improving their ability to navigate resources, as well as increasing their health knowledge, reducing their health-related stress, and reducing their overall stress related to being in a new country.

These findings are relevant in considering some of the existing literature on health knowledge in international youth populations, as well as help-seeking behaviors. As discussed in the introduction, international students are less likely to seek help for mental health conditions and were also found to have lower sexual-health literacy compared to their domestic counterparts. Our findings indicate that when international students are provided with structured education, they show a significant increase in their self-rated ability to find and access relevant school resources, as well as their health knowledge on these relevant topics.

There are some limitations to consider in our study. Of note, we did not have a formal study recruitment protocol. Researchers did provide a brief overview of the program at international student orientation sessions over the duration of the study. In addition, posters and handouts with information about the program were readily available and displayed at the Campus Health Centre. Recruitment was impacted by the COVID-19 pandemic, as researchers were unable to actively recruit participants for more than half of the study duration due to institutional COVID-19 restrictions; recruitment was largely based on word-of-mouth among the international student population. Another limitation to consider is the lack of a specific control group. Our protocol consisted of a pre-and post-intervention analysis on the same group of students. Another approach would have been to compare international students who received the educational intervention, to those who did not receive any intervention. This approach could be used to determine if stress levels, and education could have improved simply with time spent in Canada. However, the impact of this potential effect was likely limited by the relatively short pre- and post-interval of two weeks. Regardless, having a control group that does not receive the intervention would help delineate whether these issues improve with time or if the benefits are specific to the intervention. In addition, since participant responses to the survey were all self-ratings, there is certainly a possibility of bias in their self-assessment including overconfidence in their baseline knowledge. However, the pre-post design and short interval between sessions should still allow for an accurate determination of change in self-ratings due to the intervention. Finally, it is also relevant to consider the makeup of the research team as a possible limitation or barrier. Both of the lead physicians identified as male; in contrast, the nurses who provided the education all identified as female. Having a team that includes both male and female physicians and nurses and giving students the option of whom they would like to see may help students feel more comfortable in participating in discussions regarding sensitive topics. For example, a female student may feel more comfortable seeing a female physician.

In considering the future implications of this research, post-secondary institutions may consider providing routine and structured pre-arrival teaching to all international students. Providing this type of education prior to arrival may better inform students of the challenges they may face upon arriving in a new country and could also be used as an opportunity to inform students of the services they could access in the event of those challenges. These educational opportunities would likely help increase the use of resources among this population, which has been an established problem, especially in the area of mental health. Looking even more broadly, a structured intervention implemented by a multidisciplinary team could be beneficial to boosting the health literacy of all newcomer populations including immigrants and refugees. Future research can also assess how other cultural factors may affect the impacts of similar educational interventions. For example, whether the gender, or ethnicity of the providers has an impact on the measured impact or outcomes, and whether providing teaching in different languages has an impact. There would also likely be some utility in having future qualitative research which focuses more on the barriers which lead to low resource use by international students, and how to increase the use of those resources.

## Conclusions

In summary, providing international students with structured educational teaching shows significant positive increases in their self-rated health knowledge, ability to access resources, as well as reductions in various areas of stress. Post-secondary institutions should strongly consider providing structured health education to international students, as providing students with this base knowledge on key topics will help in their transition to a new country and in adapting to various cultural differences.

Given the limitations within our study of the gender and ethnicity of our physician and nurse team, future research should further assess how these factors can also play a role in providing health education to international students in order to develop education programs which students feel comfortable engaging with.

Given the significant stressors and challenges which international students face in transitioning to a new country, providing structured health education can help these students in adapting to this transition by increasing awareness and utilization of available resources and supports. As the population of international students in Canada continues to grow and provide significant contributions to our country, it is imperative to provide these students with the necessary support to help them succeed.
